# Evidence for TGF-β1/Nrf2 Signaling Crosstalk in a Cuprizone Model of Multiple Sclerosis

**DOI:** 10.3390/antiox13080914

**Published:** 2024-07-29

**Authors:** Coram Guevara, Sinay C. Vicencio, Ignacio S. Pizarro, Francisca Villavicencio-Tejo, Rodrigo A. Quintanilla, Pablo Astudillo, Estibaliz Ampuero, Rodrigo Varas, Juan A. Orellana, Fernando C. Ortiz

**Affiliations:** 1 Mechanisms of Myelin Formation and Repair Laboratory, Departamento de Biología, Facultad de Química y Biología, Universidad de Santiago de Chile, Santiago 9170022, Chile; 2 Laboratory of Neurodegenerative Diseases, Instituto de Ciencias Biomédicas, Facultad de Ciencias de la Salud, Universidad Autónoma de Chile, Santiago 8910060, Chilerodrigo.quintanilla@uautonoma.cl (R.A.Q.); 3 Instituto de Ciencias Biomédicas, Facultad de Ciencias de la Salud, Universidad Autónoma de Chile, Santiago 8910060, Chile; 4 Laboratorio Neurofarmacología del Comportamiento, Facultad de Química y Biología, Universidad de Santiago, Santiago9170022, Chile; 5 Facultad de Ciencias de la Salud, Universidad Autónoma de Chile, Santiago 8910060, Chile; 6 Departamento de Neurología, Escuela de Medicina, Facultad de Medicina, Pontificia Universidad Católica de Chile, Santiago 8330024, Chile; 7 Centro Interdisciplinario de Neurociencias, Pontificia Universidad Católica de Chile, Santiago 8330024, Chile

**Keywords:** multiple sclerosis, demyelination, neuroinflammation, oxidative stress, transforming growth factor β1 TGF-β1, Nrf2

## Abstract

Multiple sclerosis (MS) is a chronic and degenerative disease that impacts central nervous system (CNS) function. One of the major characteristics of the disease is the presence of regions lacking myelin and an oxidative and inflammatory environment. TGF-β1 and Nrf2 proteins play a fundamental role in different oxidative/inflammatory processes linked to neurodegenerative diseases such as MS. The evidence from different experimental settings has demonstrated a TGF-β1-Nrf2 signaling crosstalk under pathological conditions. However, this possibility has not been explored in experimental models of MS. Here, by using the cuprizone-induced demyelination model of MS, we report that the in vivo pharmacological blockage of the TGF-β1 receptor reduced Nrf2, catalase, and TGFβ-1 protein levels in the demyelination phase of cuprizone administration. In addition, ATP production, locomotor function and cognitive performance were diminished by the treatment. Altogether, our results provide evidence for a crosstalk between TGF-β1 and Nrf2 signaling pathways under CNS demyelination, highlighting the importance of the antioxidant cellular response of neurodegenerative diseases such as MS.

## 1. Introduction 

Multiple sclerosis (MS) is a neuroinflammatory, chronic, and degenerative disease that impacts central nervous system (CNS) function. This disease represents the leading cause of disability in young adults [1,2], being more frequent in women than in men, with a ratio of around 3:1. MS affects more than two million people worldwide, thus leading to a significant socioeconomic impact [3,4]. 

MS is characterized by the lack of myelin, a specialized membrane that enables neurons to carry out proper, fast communication. Therefore, CNS tissue features multiple demyelinated regions (i.e., loss of myelin) ubiquitously distributed, with the concomitant loss of oligodendrocytes (Ols, the myelin-forming cells at the CNS), reactive gliosis, and neuronal/axonal degeneration, commonly associated with impaired blood–brain barrier (BBB). Demyelination events are followed by neuronal degeneration, the most important cause of neurological disability in MS [5,6]. The demyelination process can be classified as primary, when it occurs by oligodendrocyte death; or secondary, when the lack of myelin is caused by infections, traumas, or axonal degeneration [7]. The leading causes of primary demyelination are associated with acute inflammatory damage to myelin or OLs and genetic vulnerability of glia (leukodystrophies) [7]. 

Although the mechanisms underlying the inflammatory process that induces demyelination are not completely understood, the current evidence indicates that there is an early event of BBB permeabilization that allows the infiltration of CD4+ T lymphocytes into the CNS, which, in turn, acts against myelin proteins and triggers the overactivation of white matter microglia and astrocytes [8]. Depending on the cytokines and factors released, microglia and astrocytes might play both neuroprotective and damaging roles in MS pathology [9]. In line with this, it has been shown that astrocytic secretion of growth factors, such as transforming growth factor β1 (TGF-β1), can exert a protective effect on demyelinating lesions by promoting remyelination [10,11].

The TGF-β pathway comprises a superfamily of cytokines that have several regulatory functions in neurogenesis, synaptogenesis, and gliogenesis [12]. TGF-β1 to -β3 have been implicated in the inflammatory process, and the TGF-β receptors I (TGF-βRI) and II (TGF-βRII) are expressed in neurons, astrocytes, OLs, and microglia [12]. In this regard, it has been shown that TGF-β family ligands are involved in inflammatory responses through the activation of microglia in several animal disease models, including stroke [13], Alzheimer’s disease (AD) [14], and MS [15]. Clinical studies have reported increased blood levels of TGF-β1 in MS patients from both relapsing-remitting and progressive forms of the disease [16], but the role of this cytokine remains elusive. As described for glial cells in demyelinating lesions, the evidence suggests that TGF-β1 might play a dual role in MS progression. In this regard, TGF-β1 might induce astrogliosis and trigger an OL-defective genetic program in MS patients [17,18], although it might also reduce the neuroinflammatory response in MS patients and diminish the activation of B-lymphocytes in the experimental autoimmune encephalomyelitis (EAE) model of MS [17,19]. It has also been reported that TGF-β1 could promote remyelination in both the EAE and toxin-induced demyelination models of MS [10].

Another common feature of MS is mitochondrial dysfunction, impairing not only redox balance and bioenergetics, but also proper neuronal function [20]. Several of these defects have been attributed to Nrf2 signaling imbalances, as they control redox balance and mitochondrial function, with potentially significant effects on the transition between the protective and detrimental neuroinflammatory status [21,22,23,24]. 

Nrf2 is a transcriptional factor that regulates the basal and stress-inducible expression of several genes containing antioxidant response elements (ARE) in their promoters [25,26]. These genes encode the expression of defensive antioxidant enzymes such as heme oxygenase-1 (HO-1), NAD(P)H quinone oxidoreductase-1 (NQO1), and glutathione S-transferase (GST), among others. Several studies have reported a close relationship between glial neuroinflammation, Nrf2-pathway activation, and the progression of the MS phenotype in MS models [27,28,29]. Moreover, several reports indicate that dimethyl fumarate—an FDA-approved drug for the treatment of MS [30]—might exert its action through Nrf2 [31,32], although the underlying mechanisms remain to be fully elucidated [33]. Importantly, a close relationship between TGF-β1 and Nrf2 signaling activation has been described in several oxidative scenarios, such as diabetes, fibrosis, cardiorespiratory dysfunction, and alcohol-induced inflammation [34,35,36,37]. However, to our knowledge, this possibility has not been tested in MS models. In the present short report, we show evidence of the crosstalk between these two pathways at the protein and functional level in a murine model of MS.

## 2. Methods 

### 2.1. Reagents and Antibodies

The reagents and antibodies used were as follows: Cuprizone (C9012-25G), DMSO, ketamine, xylazine, paraformaldehyde (P6148-500G) (from Sigma-Aldrich, Darmstadt, Germany), Galunisertib (GAL; HY-13226, MedchemExpress, Princeton, NJ, USA), normal goat serum (NGS; 50062Z, Life Technologies, Carlsbad, CA, USA), 0.5% Triton X-100 (E5111) and mouse anti-CC1 antibody to detect oligodendrocytes (MA1 25884; 1:300) (Thermo Fisher Scientific, Waltham, MA, USA), chicken anti-myelin basic protein to detect myelin (MBP; 1:800; Invitrogen, Waltham MA, USA), rabbit anti-phosphorylated Smad2 (138D4; 1:1000), anti-Smad 2/3 (D7G7, 1:1000), rabbit anti-Nrf2 (D1Z96; 1:1000 WB, 1:200 IF) (Cell Signaling, Danvers, MA, USA); mouse anti-TGF-β1 (sc-130348; 1:800); and rabbit anti-Iba1 (sc-32725; 1:50) (from Santa Cruz, TX, USA). Alexa Fluor 488–conjugated donkey antibody against chicken IgG (1:500), Alexa Fluor 555 (1:500), Tris-HCl (BM 2000), 1% Triton-X 100 and 1 mM etylenediaminetetraacetic acid (EDTA, ED0760) (Winkler, IN, USA) and protease inhibitor (04906845001; 04693159001) (Roche, Basel, Switzerland).

### 2.2. Animals 

Twelve-week-old C57BL/6 female and male mice from the animal facility of the University of Santiago de Chile were used in this study. The mice were housed in plastic cages in a temperature-controlled (24 °C) and humidity-controlled vivarium under a 12 h light/dark cycle (lights on 8:00 AM, with ad libitum access to food and water), and the experiments were performed during the light period. The animal experimentation and protocols were approved by the Bioethical Committee for Animal Experiments of the University of Santiago de Chile (protocol number 319/2023) in accordance with Guide for the Care and Use of Laboratory Animals (National Institutes of Health, Bethesda, MD, USA). 

### 2.3. Experimental Model 

#### 2.3.1. Cuprizone Administration 

The MS murine model was carried out via toxin-induced demyelination with cuprizone (CPZ), a widely used MS experimental model based on the feeding of animals with CPZ. Cuprizone is a copper chelator that induces demyelination by toxicity of OLs ubiquitously in the CNS and is, therefore, widely used to generate models of central demyelinating diseases such as MS [28,38,39]. The model consists of inducing demyelination in animals by having them ingest cuprizone—mixed in with the food at 0.25%—over 5 weeks, the time necessary to generate demyelination [40].

#### 2.3.2. TGF-β1R-Blocker Administration 

**Figure 1:**
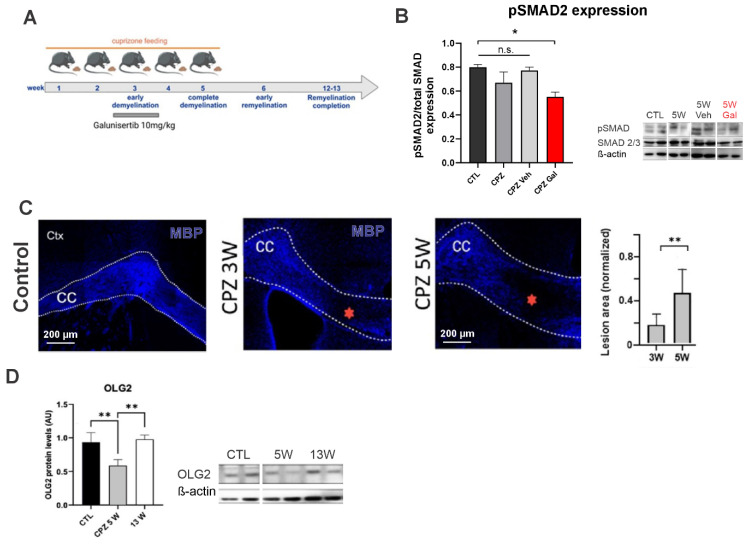
Experimental model of MS with cuprizone-induced demyelinated lesions. (**A**) Wild-type mice were fed cuprizone (CPZ, 0.25%) for 5 weeks. The treated group received the TGF-β1 receptor blocker galunisertib (GAL, 10 mg/kg) daily during the third week of CPZ feeding. Note the expected reduction in pSMAD in the mice treated with the TGF-β1 receptor blocker GAL compared to the control animals (**B**) (*) *p* < 0.05. (**C**) Immunofluorescence of 75-µm-thick callosal coronal sections from the control mice or mice fed with 0.25% cuprizone over 3 and 5 weeks (CPZ 3W and 5W, respectively). Dotted lines indicate the corpus callosum (CC). Demyelinated areas, recognized by the lack of myelin basic protein (MBP) expression (blue), are indicated by red asterisks. The summary of the normalized lesion size is also shown (plot, Mann–Whitney test, (**) *p* < 0.01, *n* = 3–4 animals). (**D**) Protein levels for the oligodendroglial marker OLG2 at basal line (Ctl) and weeks 5 and 13 (CPZ 5W and 13W, respectively) of the cuprizone model (Kruskal–Wallis test, (**) *p* < 0.01, *n* = 3–4; representative images of western blot membranes from two mice are shown).

To determine the contribution of TGF-β1 to the early stages of demyelination, TGF-β1 receptor was pharmacologically blocked in the CPZ animals. For this, the TGF-β1 receptor blocker galunisertib (GAL) [41,42,43] was orally administered in the drinking water (10 mg/kg, diluted in 0.2% DMSO and 10% glucose serum) daily during the third week of the CPZ feeding (i.e., at the beginning of demyelination; Figure 1).

The animals were randomly divided into the following four experimental groups: Group 1: mice fed with conventional chow food (CTL); Group 2: mice fed with CPZ (CPZ); Group 3: mice fed with CPZ and GAL (CPZ + GAL); and Group 4: mice fed with CPZ and treated with vehicle (CPZ + VEH).

### 2.4. Immunostainings

After 5 weeks, the mice were perfused under deep anesthesia (ketamine/xylazine 75/7.5 mg/kg, respectively) with 4% paraformaldehyde. Brain samples were stored at 4 degrees in PBS (1X) for immunofluorescence assays.

Coronal brain sections (75 µm width) were blocked with PBS containing 4% normal goat serum (NGS) and 0.5% Triton X-100 for 2 h at room temperature. Brain slices were incubated with primary antibodies diluted in the blocking solution (PBS containing 2% NGS and 0.2% Triton X-100) overnight at 4 °C. The following primary antibodies were used: chicken anti-myelin basic protein (MBP; 1:800 Invitrogen) to detect myelin, mouse anti-TGF-β1 antibody (1:100), rabbit anti-Nrf2 (1:400), rabbit anti-Iba1 (1:500) to detect microglia/macrophage, and mouse anti-CC1 (1:300). The slices were then incubated for 2 h at room temperature with Alexa Fluor 488–conjugated donkey antibody against chicken IgG (1:500) or Alexa Fluor 555 (1:500). Images were acquired via confocal microscopy with a Zeiss LSM510 confocal microscope (LSM510 software version 3.2; Carl Zeiss, Oberkochen, Baden-Württemberg, Germany) with the following parameters: acquired at 20X (NA 0.8 objective) under 488/555 nm excitation in Z-stack, 7–8 µm width. The acquired images were then reconstructed in Z-projections averaging 7 to 10 optical sections per sample (49 to 80 µm width). Image analysis was performed with ImageJ software version 1.54.

### 2.5. Western Blot Analysis

White matter was homogenized in 10 mM Tris-HCl at pH 7.4, 150 mM NaCl, 1% Triton-X 100, and 1 mM ethylenediaminetetraacetic acid (EDTA) containing protease inhibitors. Lysates were clarified by centrifugation at 8000× *g* (4 °C) for 20 min, and the supernatants were collected and normalized for protein concentration. Proteins were separated by 10% and 15% sodium dodecyl sulfate–polyacrylamide gel electrophoresis (SDS-PAGE) and transferred onto polyvinylidene difluoride membranes (Immobilon-P, Millipore, Burlington, MA, USA). After blocking with PBS containing 5% skim milk and 0.05% Tween 20, the membranes were incubated with primary antibodies overnight at 4 °C, followed by incubation with an HRP-conjugated secondary antibody for 2 h at room temperature. The following antibodies were used: mouse anti-TGF-β1 and rabbit anti-Nrf2. Horseradish-peroxidase-conjugated anti-rabbit IgG antibody was used as the secondary antibody (Cell Signaling Technology). Immunoreactive bands were detected using a fluorescence-conjugated secondary antibody and an enhanced chemiluminescence (ECL) system (WBKLS0100, Millipore). They were visualized on a LAS-4000 imaging system (Fujifilm). The protein bands were quantified using the ImageJ software. 

### 2.6. ATP Bioluminescence Assay 

For an indirect determination of mitochondrial function, ATP concentrations (pMol/g) were measured in fresh corpus callosum tissue from the animals fed with cuprizone for 3 and 5 weeks and in animals treated with GAL and vehicle for 7 days in week 3 of cuprizone feeding (see Figure 1). Briefly, the tissue was mechanically homogenized and, afterwards, the total protein was quantified (protein BCA kit, Thermo), and the ATP levels were determined by using an ATP bioluminescence assay following the manufacturer’s instructions (ATP Determination Kit, Invitrogen A22066).

### 2.7. Locomotor Rotarod Test

The rotarod test was employed to assess locomotor function [44]. The mice were placed in individual cylinder lanes of the rotarod apparatus (Rotarod 755, IITC, Lifescience). The speed of the cylinder was linearly increased from 4 rpm to 40 rpm for 300 s while time-to-fall on the rotarod was measured. Three repetitions (7-min rest intervals) were carried out during each evaluation day. This evaluation was performed for two consecutive days. Before starting the test, the animals underwent a period of habituation in the equipment. The basal line of the locomotor function was obtained one week before the beginning of the CPZ feeding.

### 2.8. Evaluation of the Cognitive Function

To assess the cognitive function of the mice, we evaluate the spatial memory by performing the novel object recognition test (NOR) [45,46], which consists of placing the animal in a space with two identical objects for 10 min (training) and, 24 h later, changing one of the objects. The time in which the animal interacts with each object is measured The time that the animal spent with the new object is subtracted from the time spent with the old object and then divided by the total interaction time, which yields the interaction index, which is then finally compared. The mice were habituated daily during the 3 days prior to performing the test. The basal line of the interaction index was obtained one week before the beginning of the CPZ feeding. All tests and data were collected using AnyMaze software version 7.44, (Stoelting 60000D).

### 2.9. Statistical Analysis

All data are presented in box and whisker plots (5–95 percentile), except for the ATP measurements, which are shown as mean ± SEM. After the test for normality, data from two groups were compared using the Mann–Whitney test, while three or more groups were compared by using one-way ANOVA or the non-parametric equivalent Kruskal–Wallis test, according to the data structure. The post hoc test is indicated when it corresponded. Statistical significance was defined as *p* = 0.05. All statistical analyses and plots were performed using GraphPad Prism 8.0 software.

## 3. Results

### 3.1. Blockage of the TGF-β1 Receptor Reduces Both Nrf2 and TGF-β1 Protein Levels in the CPZ Model of MS

Nrf2 is one of the major endogenous factors controlling the antioxidant cellular response in mammals [21,22,23,24]. Since demyelination is accompanied by a strong oxidative and neuroinflammatory environment [8], we studied the expression of this factor in demyelinated conditions. To achieve this, we implemented the cuprizone (CPZ) model of demyelination (Figure 1), which consists of triggering demyelination in CNS by feeding animals with CPZ. This copper chelator specifically kills oligodendrocytes at the applied dose (0.25%). One advantage of this model is the stereotyped fashion of the demyelination process, where demyelination insult begins at the 3rd week (3W) of CPZ feeding, ending around the 5th week (5W), when CPZ feeding is stopped (Figure 1A). From weeks 6 to 13, a spontaneous process of myelin repair (i.e., remyelination) occurs (Figure 1A). Since we sought to study the initial demyelination process, we evaluated the experimental readouts at 3W and 5W after the beginning of the CPZ treatment. Of note, galunisertib (GAL) treatment partially reduced pSmad2 levels, concomitant with the expected TGF-β1 inhibition (Figure 1B). We corroborated that CPZ feeding induced demyelination in the CNS, particularly in the corpus callosum regions (Figure 1C). As expected, the demyelinated regions (i.e., lesions) were larger at 5W compared with those found at 3W of CPZ feeding (Figure 1C). Concomitantly, the measurements of the OLG2 protein levels—a marker of the OL lineage—dropped at 5W and were recovered at 13W, when remyelination was expected to be completed (Figure 1D), validating our in vivo demyelination murine model.

**Figure 2:**
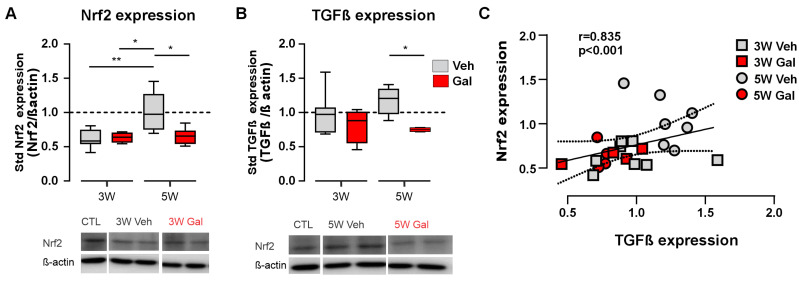
TGF-β1 receptor 1 blocking reduces both Nrf2 and TGF-β1 protein levels in the CPZ model of MS. (**A**,**B**) Protein levels in respect to the control and normalized by β-actin for Nrf2 (**A**) and TGF-β1 (**B**) in the corpus callosum of the mice fed with CPZ over 3 or 5 weeks (3W and 5W) treated with galunisertib (GAL, 10 mg/kg, red blocks) or vehicle (Veh, gray blocks). Representative images of western blot membranes from two mice are shown (bottom). Kruskal–Wallis test, Dunn’s multiple comparison post hoc tests (*) *p* > 0.05, (**), *p* < 0.01. (*n* = 4–8 mice). (**C**) Correlation between protein levels for TGF-β1 and Nrf2 at the different time points and treatments (r = 0.835, *p* < 0.001, Spearman test).

We evaluated the expression of Nrf2 in the CNS tissue of the CPZ mice to determine whether this protein was differentially expressed during the progression of demyelination. The protein concentrations of Nrf2 were determined by western blot analysis in the corpus callosum tissue homogenates from the animals fed with cuprizone for 3 and 5 weeks and treated with galunisertib (GAL) or vehicle (VEH) during the 3rd week (Figure 1A). We confirmed the action of GAL as a TGF-β1R blocker by quantifying the phosphorylated form of the SMAD2 protein (pSMAD2), a conserved element of the TGF-β1R activation pathway [47,48]. We then measured the relative expression of Nrf2. Nrf2 expression was reduced at 3W to approximately 61% of the control values, while, at 5W after the beginning of the CPZ treatment, the Nrf2 levels were recovered (Figure 2A). However, Nrf2 failed to recover from the lower expression when evaluated at 5 weeks in the GAL-treated group. No changes were observed when quantified at 3 weeks, suggesting a role of TGF-β1R signaling in the recovery of Nrf2 expression at 5 weeks of CPZ feeding (Figure 2A). These results suggest that the blocking of TGF-β1 signaling at the beginning of demyelination (third week) impairs the recovery of Nrf2 levels in the corpus callosum. 

In addition to the possible crosstalk between TGF-β1 and Nrf2 signaling, it has been reported that TGF-β1 signaling might control its own expression (i.e., TGF-β1 might promote the expression and release of more TGF-β1, resembling feedforward control) [49]. Therefore, we investigated this possibility under demyelinated conditions. We observed conserved TGF-β1 expression at the third week of cuprizone feeding, both with and without the administration of the TGF-β1 receptor blocker, compared to the control levels (Figure 2B). However, in the fifth week, the CPZ-treated mice showed an increased expression of TGF-β1, which was blocked by GAL (Figure 2B). These results are in concordance with previously published findings supporting a mechanism for TGF-β1-induced TGF-β1 release; moreover, more importantly, it also provides evidence to support a possible crosstalk between these two pathways in the CPZ model of MS, in which the TGF-β1 receptor activation might induce the observed recovery of Nrf2 protein expression at the peak of demyelination. Indeed, we also found a highly significant correlation between the expression levels of TGF-β1 and Nrf2 across treatments and the different time points (Figure 2C, r = 0.835, *p* < 0.001, Spearman test), further supporting the close relationship between these two factors. 

### 3.2. TGF-β1 Receptor Blocking Reduces ATP Production and Catalase Expression during Demyelination

**Figure 3:**
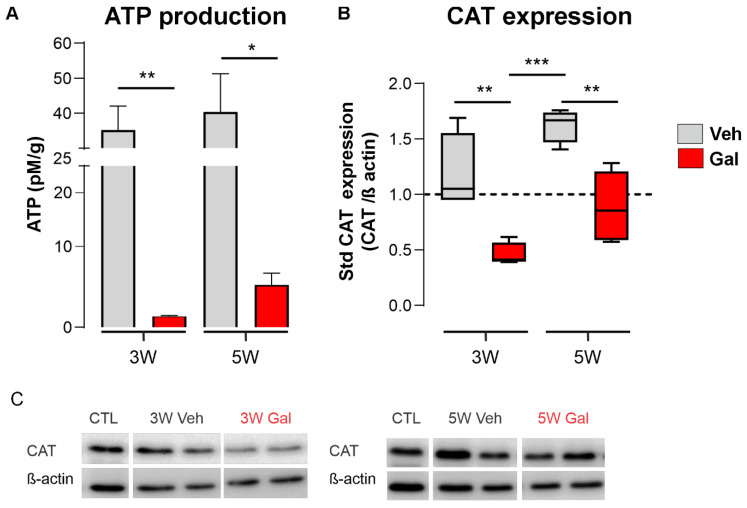
TGF-β1 receptor 1 blocking reduces both ATP production and catalase protein levels in the CPZ model of MS. (**A**) ATP bioluminescence quantification in the corpus callosum of the mice fed with CPZ over 3 or 5 weeks (3W and 5W) treated with galunisertib (GAL, 10 mg/kg, red blocks) or vehicle (Veh, gray blocks). (**B**) Protein levels in respect to the control and normalized by β-actin for catalase (CAT) in the corpus callosum of the mice fed with CPZ over 3 or 5 weeks (3W and 5W) treated with galunisertib (GAL, 10 mg/kg, red blocks) or vehicle (Veh, gray blocks). (**C**) Representative images of western blot membranes from two mice are shown. Data in A are expressed as the mean ± standard error. Kruskal–Wallis test, Dunn’s multiple comparison post hoc tests (*) *p* < 0.05; (**), *p* < 0.01: (***), *p* < 0001. (*n* = 3–6 mice for ATP measurements, *n* = 4–6 mice for CAT levels).

The Nrf2-dependent cellular antioxidant response involves the activation of many antioxidant enzymes and the preservation of mitochondria function, the latter allowing cells to maintain ROS and calcium management, as well as ATP synthesis. In this regard, we explored the effects of GAL treatment on these two major readouts of Nrf2 activation, namely, ATP production and the relative levels of catalase (CAT), one of the main effectors of the enzymatic antioxidant response. We measured the levels of ATP and CAT expression in the homogenized corpus callosum tissue from animals that had been fed with CPZ and treated with GAL or vehicle (Veh) (Figure 3). Blocking the TGF-β1 receptor significantly decreased both the levels of ATP and the relative expression of CAT in the corpus callosum of the CPZ-treated mice. Interestingly, the effect of GAL was significant at 3 and 5 weeks. These results suggest that TGF-β1 signaling might have a protective role on the mitochondrial function and the cellular antioxidant response during demyelination (Figure 3).

### 3.3. TGF-β1 Protects Animals from Locomotor and Cognition Impairment Induced by Cuprizone

**Figure 4:**
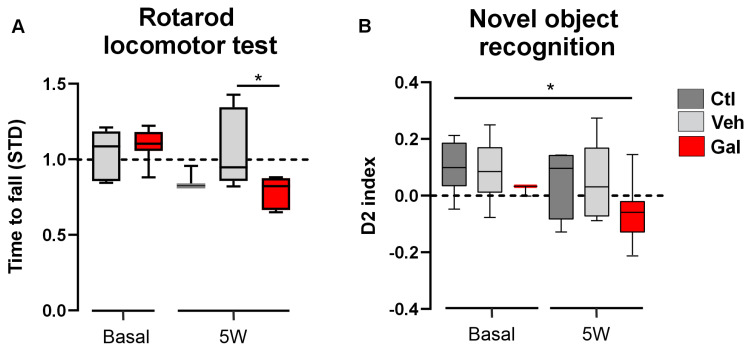
TGF-β1 receptor 1 blocking reduces the locomotor performance and the cognitive function in the CPZ animal model of MS. (**A**) Summary of the standardized time-to-fall in the rotarod apparatus for mice before (basal) and after being fed with CPZ for 5 weeks with no treatment (control, Ctl, dark-gray blocks) or treated with galunisertib (GAL, 10 mg/Kg, red blocks) or vehicle (Veh, light-gray blocks). Note that all data are normalized in respect to the basal value with no treatment. (**B**) Summary of the interaction (D2) index with the novel object for mice before (basal) and after being fed with CPZ for 5 weeks with no treatment (control, Ctl, dark-gray blocks) or treated with galunisertib (GAL, 10 mg/Kg, red blocks) or vehicle (Veh, light-gray blocks). Kruskal–Wallis test, Dunn’s multiple comparison post hoc tests, (*) *p* < 0.05 (*n* = 3–8 mice for locomotor test, *n* = 3–7 mice for NOR test).

In MS, early events of demyelination have been extensively associated with neurological damage. In patients, major hallmarks of damage include motor, locomotor, and cognitive impairment [50,51,52,53]. Therefore, we investigated whether the observed effects of GAL treatment during demyelination induction might correlate with the locomotor performance and cognitive function of the CPZ mice after the completion of the demyelinated phase (i.e., at 5 weeks). Rotarod and NOR tests were carried out on the CPZ mice treated with GAL or vehicle (Veh). We observed no impairment change in either the locomotor or cognitive function at 3W or 5W in the control mice (i.e., basal measurements or CPZ mice treated with Veh, Figure 4). Interestingly, the mice presented a significant decrease in the time-to-fall time in the rotarod test following treatment with the TGF-β1R blocker (Figure 4A), indicative of locomotor impairments, and showed a significant reduction in the interaction index in the cognitive NOR test (Figure 4B), an indirect measure of spatial memory performance in animals. Taken together, these results suggest that TGF-β1 signaling plays a protective role in both the locomotor and cognitive performance of CPZ mice.

## 4. Discussion 

**Figure 5:**
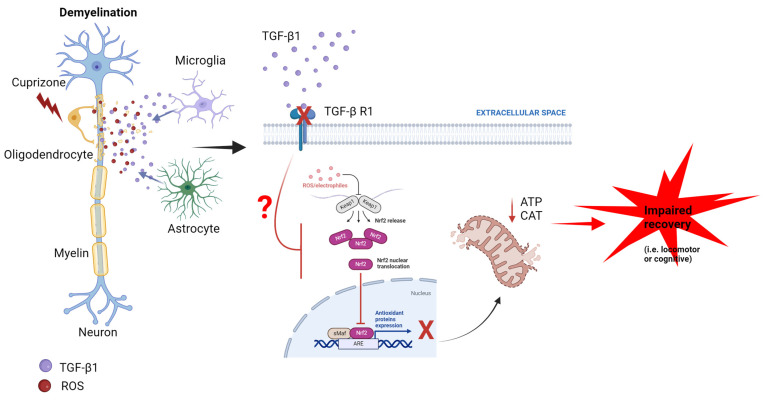
Putative mechanisms of the TGF-β1/Nrf2 signaling crosstalk in demyelinated lesions. Under demyelination by cuprizone, TGF-β1 expression increases, most likely promoting astrocyte and microglia TGF-β1 release. The activation of the TGF-β1 receptor will, in turn, activate an Nrf2-dependent antioxidant response, such as enhanced catalase (CAT) expression and ATP synthesis. Thus, blocking the TGF-β1 receptor reduces this response by mechanisms to be determined (?), triggering a failure in antioxidant-related recovery (for instance, locomotor and cognitive function). This figure was created in Biorender.

Here, we provide evidence that Nrf2 protein expression is reduced upon TGF-β1 receptor blocking in the demyelination phase of the MS cuprizone model. Additionally, we found that both TGFβ-1 and catalase expression, along with ATP production, were reduced by blocking the TGFβ-1 receptor, while locomotor function and cognitive performance were also diminished (Figure 5).

The TGF-β1 and Nrf2 proteins play a fundamental role in the different inflammatory processes linked to neuroinflammatory/neurodegenerative diseases such as multiple sclerosis [10,33,34,54,55,56]. Here, we have reported that Nrf2 protein levels decrease at the beginning of demyelination (week 3 of the CPZ model) and then recover when demyelination is complete (week 5), suggesting an activation of the pathway and, therefore, an expected compensatory response [10]. The block of the TGF-β1 receptor abolished this recovery, highlighting the protective role of TGF-β1 in the progression of the demyelinated lesions. Reinforcing this notion, we found a close correlation between the levels of both proteins. In this regard, it has been reported that both Nrf2 and TGF-β1 are upregulated in inflammatory environments, mitigating oxidative/inflammatory damage in different experimental settings [57,58,59]. For example, in experimental models of liver fibrosis, TGF-β1 suppressed the Nrf2-dependent expression of NQO1 (a major antioxidant enzyme) [57,60], and the inhibition of Nrf2 activation by dimethyl fumarate inhibited the profibrotic action of TGF-β1 [58,59]. These studies support crosstalk of the pathways in both directions, i.e., the control of TGF-β1 on Nrf2 signaling and vice versa. In this sense, our reported reduction in TGF-β1 protein levels when blocking the TGF-β1 receptor might be explained by a TGF-β1-induced TGF-β1 release [49], but also by a putative Nrf2-dependent control. Evidence from rat tubular epithelial cells [61] and, closer to our model, from experimental traumatic brain injury (TBI) [62], has also demonstrated a crosstalk between these two signaling molecules; however, to our knowledge, this possibility has not been explored in experimental MS before the present report.

Another aspect highlighted here is the putative role of TGF-β1 on mitochondrial function, ATP levels, and catalase protein expression. Energy production occurs preferentially in mitochondria. In this regard, a recent report has demonstrated that, under demyelinated conditions, there was a recruitment of mitochondria from the neuronal soma to the demyelinated regions of the axon, suggesting a compensatory mechanism in response to the energy failure resulting from demyelination (i.e., the axonal response of mitochondria to demyelination [ARMD]) [28]. Thus, our results showing increased ATP production in the demyelinated callosal fibers might be explained by the presence of an ARMD-like mechanism. 

The antioxidant Nrf2-dependent response is associated with an increased expression and activity of antioxidant enzymes such as catalase, along with an enhanced mitochondrial function, including ROS reduction and increased ATP synthesis [63]. Our results might be partially explained by these cellular mechanisms since the treatment with galunisertib reduced Nrf2 and catalase expression, as well as ATP production; however, we observed a significant reduction in Nrf2 protein levels only at the fifth week, while the catalase and ATP measurements were affected at both the third and the fifth weeks. The latter raises the possibility that TGF-β1 exerts a protective role (i.e., increasing the catalase activity and ATP production) through different pathways depending on the stage of the demyelination process. Then, TGF-β1 might preferentially act through an Nrf2-independent manner at the early demyelination stages—namely, at the onset of the oxidative and inflammatory responses—for instance, by activating another factor involved in the antioxidant/anti-inflammatory cellular response. Future studies are needed in order to determine the TGF-β1-dependent mechanisms at the different stages of the demyelinated process. 

Demyelination diseases such as MS are characterized by several degrees of cognitive and locomotor impairment [1,4,7], thus, experimental MS models normally display some of these disabilities. In the case of the CPZ model of MS, these characteristics generally appear after the initial demyelination stages [38,39,41,64]. Interestingly, the cuprizone-treated mice of our study did not show locomotor or cognitive damage at 5 weeks, however, when they were treated with the TGF-β1 receptor blocker, both assessments where reduced; therefore, although preliminary, these results allow for the proposal of a protective role of TGF-β1 on the at least these two functional parameters in the CPZ model. Further investigations using a longitudinal experimental design will allow us to characterize the role of TGF-β1-dependent pathways on the motor and cognitive characteristics of the CPZ model of MS.

Our investigation provides evidence of Nrf2 and TGF-β1 crosstalk in a demyelination mouse model (Figure 5); however, the cellular mechanisms underlying this phenomenon are still unknown. A limitation of this study is the lack of identification of the cellular types involved in TGF-β1/Nrf2 signaling. Considering the complex degenerative and inflammatory scenario composed of pro- and anti-inflammatory reactive astrocytes, at least two different populations of microglia (M1- and M2-like), oligodendrocyte precursors, mature oligodendrocytes, and damaged neurons, etc., we will require a completely new study using new experimental tools to answer this question. For instance, in our hands, immunofluorescent Nrf2 protein expression was observed only in astrocytes and microglia (no stain in oligodendrocytes, Supplementary Figure S1), suggesting that astrocytes and microglia would be the principal cells involved in the Nrf2-dependent response. However, the Nrf2 pattern of expression (“puncta-like”) precludes more definitive conclusions with the present experimental approach.

In summary, our results provide evidence for a crosstalk between TGF-β1 and Nrf2 signaling pathways under CNS demyelination, highlighting the importance of the oxidative and inflammatory cellular management in neuroinflammatory-associated diseases such as MS. 

## Data Availability

The original contributions presented in the study are included in the article/Supplementary Material, further inquiries can be directed to the corresponding authors.

## Supplementary Materials

The following supporting information can be downloaded at: https://www.mdpi.com/article/10.3390/antiox13080914/s1, Figure S1. Immunofluorescence of Nrf2 protein in cuprizone-demyelinated lesions.
