# Supplementary material for: Evidence for TGF-β1/Nrf2 Signaling Crosstalk in a Cuprizone Model of Multiple Sclerosis
Source: Antioxidants (Basel). 2024 Jul 29;13(8):914. doi: 10.3390/antiox13080914 (PMC11351764; doi:10.3390/antiox13080914)
Supplement: Supplementary file 1 [file antioxidants-13-00914-s001.zip › antioxidants-3131429-supplementary.pdf]

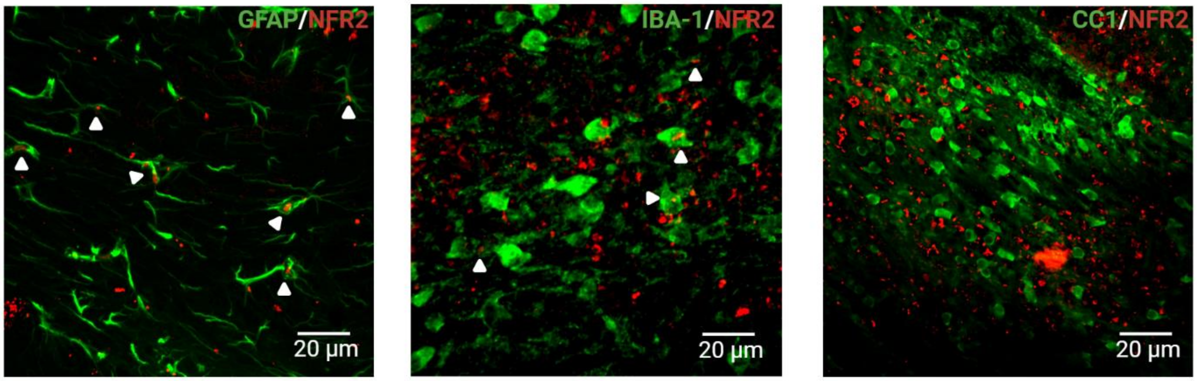

Figure S1. Nrf2 protein expression in cuprizone-demyelinated lesions. Immunofluorescence of 75 µm-thick callosal coronal sections from demyelinated mice fed with 0.25% cuprizone (3 weeks). Astrocytes, microglia/macrophages and oligodendrocytes were identified by GFAP (green, right panel), Iba1 (green, middle panel) and CC1 (green, left panel), respectively. White arrowheads indicate Nrf2 puncta (red) co-stained with cell markers (green). Note the "puncta-like" pattern of Nrf2-protein staining (N=2-3 animals)
